# A Novel *CAPN1* Mutation Causes a Pure Hereditary Spastic Paraplegia in an Italian Family

**DOI:** 10.3389/fneur.2019.00580

**Published:** 2019-06-05

**Authors:** Stefano Cotti Piccinelli, Maria T. Bassi, Andrea Citterio, Fiore Manganelli, Stefano Tozza, Filippo M. Santorelli, Serena Gallo Cassarino, Filomena Caria, Enrico Baldelli, Anna Galvagni, Lucio Santoro, Alessandro Padovani, Massimiliano Filosto

**Affiliations:** ^1^Unit of Neurology, Center for Neuromuscular Diseases, ASST Spedali Civili and University of Brescia, Brescia, Italy; ^2^Laboratory of Molecular Biology, Scientific Institute IRCCS E. Medea, Lecco, Italy; ^3^Department of Neurosciences, Reproductive Sciences and Odontostomatology, University Federico II of Naples, Naples, Italy; ^4^Unit of Molecular Medicine, IRCCS Foundation Stella Maris, Pisa, Italy

**Keywords:** HSP, hereditary spastic paraplegia, ataxia, *CAPN1*, calpain-1, SCA, spinocerebellar ataxia

## Abstract

*CAPN1* encodes calpain-1, a large subunit of μ-calpain, a calcium-activated cysteine protease widely present in the central nervous system. Mutations in *CAPN1* have recently been identified in a complicated form of Hereditary Spastic Paraplegia (HSP) with a combination of cerebellar ataxia and corticomotor tract disorder (SPG76). Therefore, *CAPN1* is now considered one of those genes that clinically manifest with a spectrum of disorders ranging from spasticity to cerebellar ataxia and represent a link between Spinocerebellar Ataxia and HSP, two groups of diseases previously considered separate but sharing pathophysiological pathways. We here describe clinical and molecular findings of two Italian adult siblings affected with a pure form of HSP and harboring the novel homozygote c.959delA variant (p.Tyr320Leufs^*^73) in the *CAPN1* gene. Although the reason why mutations in *CAPN1* may cause heterogeneous clinical pictures remains speculative, our findings confirm that the spectrum of the *CAPN1*-linked phenotypes includes pure HSP with onset during the third decade of life. Further studies are warrantied in order to clarify the mechanism underlying the differences in *CAPN1* mutation clinical expression.

## Introduction

Hereditary spastic paraplegias (HSP) are a heterogeneous group of genetically inherited diseases characterized by weakness and spasticity in the lower limbs, which may or may not be associated with other neurological symptoms (1).

The prevalence of the different forms of HSP has been estimated around 1.8 per 100,000, although some studies predicted a prevalence of between 2 and 10 subjects per 100,000 depending on the considered populations (2–5).

Based on the phenotype, HSP can be classified into pure and complicated forms (6).

Pure forms are characterized by signs of involvement of the pyramidal tract, such as weakness, spasticity, and brisk deep tendon reflexes at lower limbs with extensor plantar response. Hypertonic bladder and various grade of deep sensory impairment are also possibly associated (7).

Complicated forms present with a more heterogeneous phenotype, in which the classic spastic paraparesis/plegia is accompanied by various neurological and non-neurological disturbances, including cerebellar dysfunction, intellectual disability, axonal, or demyelinating peripheral neuropathy, seizures, extrapyramidal features, eyelid ptosis, ophthalmoplegia, opthalmological abnormalities, facial dysmorphism, and foot deformities (8, 9).

The age of onset may vary and both early- and late-onset forms have been described. Most HSP phenotypes become clinically evident between adolescence and the third decade of life (1).

HSP are also genetically heterogeneous conditions. They can be inherited in an autosomal- dominant (AD-HSP), autosomal-recessive (AR-HSP) or X-linked (XL-HSP) fashion. Maternal inheritance is also possible, despite very rarely, usually in complicated HSP phenotypes suggesting an underlying mitochondrial DNA defect (10).

To date, more than 80 types of HSP have been genetically defined by linkage analysis and identification of HSP-related gene variants (11).

Some genes are associated with pure or complicated HSP, while other genes are linked to both forms of HSP, indicating that other modifying genetic or environmental factors can be involved in determining the disease course (10, 11).

Neuropathological studies indicated that HSP are typically characterized by a length-dependent “dying back” axonopathy, with more pronounced axonal degeneration in the distal segments of the corticospinal tract and in the proximal tract of sensory fibers (12). These findings correlate with the evidence that HSP-related genes may be involved in multiple cell pathways leading to axonal dysfunction, such as endosomal trafficking, mitochondrial regulation, lipid metabolism, and regulation of the endoplasmic reticulum (5).

Very recently, *CAPN1*, the gene encoding calpain-1, has been identified as a HSP-related gene (13–16).

Although *CAPN1* mutations usually cause an autosomal-recessive complicated form of HSP, named SPG76, few recent studies reported defects in *CAPN1* also in pure HSP subjects (16–18).

We here describe clinical and molecular findings in two adult siblings affected with an uncomplicated HSP and harboring a novel mutation in *CAPN1*.

## Patients And Methods

### Patients

A 33-year-old man (Patient 1) complained, since he was 25, of pain and yielding at the right foot after standing for a long time.

The symptoms remained stable over the years until age 29, when he began to present spasticity involving both lower limbs, with a slowly worsening course.

His 42-year-old sister (Patient 2) complained of similar symptoms since age 30.

**Figure 1:**
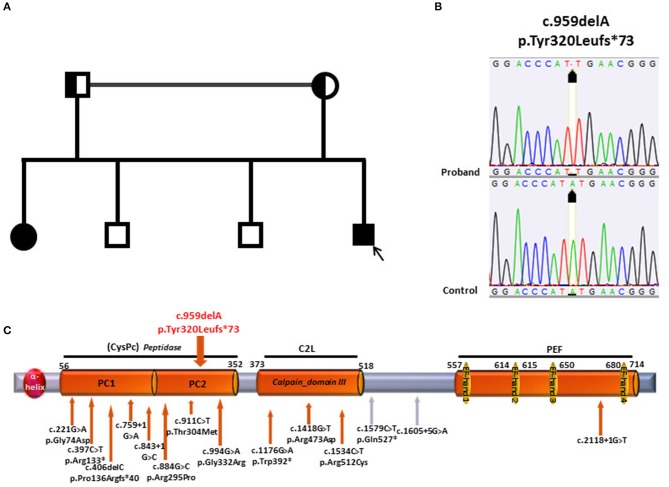
**(A)** Pedigree of the family; the patient is depicted by a black arrow. **(B)** Electropherograms of *CAPN1* sequence in patient and control. **(C)** Structure of *CAPN1* protein, Calpain 1, its domains with location of the mutations previously identified (under the gray bar) and the mutation identified in the current study (on top of the gray bar). At the N-terminus of the protein, an a-helix domain is followed by two Protease core domain, 1 and 2 (PC1and PC2), constituting the Peptidase- Cystein Protease core Peptidase (CysPC); the Calpain domain III- also known as C2-like Ca^2+^^−^Binding domain (C2L) precedes the PEF (Penta EF-hand) domain at the C terminus containing 5 EF-hand motifs.

Two other brothers of age 40 and 36, as well as the 71 year-old father and the 68-year-old mother were asymptomatic and were not examined. Parents were consanguineous (first cousins) (Figure 1A).

#### Patient 1

In the past medical history, congenital thyroid hemi-hypoplasia and levothyroxine intake were noticed.

Neurological examination disclosed severe spastic gait with increased muscle tone in the lower limbs, very brisk ankle jerks, and knee tendon reflexes, ankle clonus and Babinski sign. No tactile, stocking pin-prick, vibratory, and proprioception sensory reduction was observed. Romberg sign was absent. Cerebellar signs were absent. General physical examination was normal.

Routine laboratory tests (including blood cell count, blood glucose, vitamin B12 and folate) were in the normal range. Brain and spinal cord MRI was normal. Nerve conduction studies and needle electromyography were normal.

Motor evoked potentials were markedly slowed while sensory evoked potentials showed a mild increase of central conduction time from lower limbs.

After clinical evaluation, the patient started neuromotor rehabilitation treatment.

#### Patient 2

Her past medical history was unremarkable. She had two healthy children. Neurological examination did not differ from that of the younger brother.

Routine laboratory tests (including blood cell count, blood glucose, vitamin B12, and folate) were in the normal range. EMG-ENG and brain MRI findings were normal. Spinal cord MRI showed some disc protrusions without signs of myelopathy.

Motor evoked potential showed decreased amplitude and increased latency at four limbs with increased central conduction time.

### Genetic Analysis

The study was conducted in accordance to the ethical standards of the Declaration of Helsinki (1964).

After obtaining an informed consent from the probands and parents, their DNA was extracted from peripheral blood using GenElute Blood Genomic DNA Kit (Sigma St.Louis, Missouri, USA).

Libraries were prepared using a custom gene panel from Agilent (Santa Clara, California, USA), which enables the capturing of 202 genes known to cause hereditary spastic paraparesis and other motor neuron disorders (gene list is provided as Supplementary Material). The coding regions and flanking intronic regions of the 202 genes were enriched using the SureSelect XT Target Enrichment System from Agilent (Santa Clara, California, USA), following the manufacturer's protocol. Sequencing, was performed on the Illumina Next-Seq500 platform.

After capturing enrichment and sequencing, data were aligned to the reference sequence of the human genome (University of California Santa Cruz (UCSC) hg19/GRCh37) with BWA (the Burrows-Wheeler Alignment algorithm) and variant were called with GATK through the BaseSpace app, BWA Enrichment v.2.1.0 (Illumina, California, USA). Called variants were annotated with ANNOVAR (Wang Genomics Lab 2010-2019).

In a diagnostic setting, variants were filtered for allele frequencies <1% in the Exome Aggregation Consortium (ExAC) data set (http://exac.broadinstitute.org/) and basing on their type and genomic localization, thus synonymous and intronic variants were discarded.

On average, 98.23% and 99.5 % of bases were covered by at least 10 and 20 sequence reads, respectively. The mean read depth of the targeted regions was 1245.34X. We used Polyphen2 and SIFT to assess the functional effects of the variants. After filtering, we performed Sanger sequencing to confirm the variants detected through targeted sequencing analysis.

## Results

Genetic analysis in patient 1 showed the presence of the homozygous c.959delA variant (p.Tyr320Leufs^*^73) in the *CAPN1* gene (Figure 1B). The single base deletion leads to a frameshift with a stop codon 73 amino acid residues downstream. The protein truncation occurs within the Cysteine Protease Core domain (CysPC) located in the N-terminus of the protein (19) (Figure 1C).

The premature truncation leads to a putative protein missing the Ca-binding domains typical of calpains, i.e., Calpain domain III, also known as C2-like Ca2+-binding domain (C2L), and the PEF (Penta EF-hand) domain at the C terminus containing 5 EF-hand motifs. This modification is likely to lead to a loss of protein function.

Segregation analysis was then conducted on proband's family members and the same homozygous variant was identified in the affected sister. As expected, both parents were heterozygous carriers. The analysis was not conducted on the two healthy brothers.

## Discussion

Calpain-1 is one of the two major isoforms of calpains, together with calpain-2 (15, 19, 20).

Calpain-1 and−2 have opposite functions within the CNS, due to their associations with different signaling cascades (19). Many experimental studies have suggested that the activation of calpain-1, as opposed to activation of calpain-2 which is neuro-damaging, may promote neuroprotection and synaptic plasticity processes (15, 19, 20).

In flies, worms, mice and zebrafish knockdown or knockout models, the absence of Calpain-1 orthologs led to neurodegeneration and motor impairment (15, 16). Particularly, in *CAPN-1* knockout zebrafish, brain and spinal cord microtubule network appeared disorganized and regions with abnormal accumulation of tubuline close to regions with complete depletion were observed (16). Impaired neuron migration and positioning, increased neurotoxicity and disruption of brain development were described, thus supporting a neuroprotective role for calpain-1 (16).

On the other hand, some findings have suggested that overactivation of calpains, including Calpain-1, might be detriment and contribute to the pathogenesis of traumatic brain injury and Alzheimer's disease although it have been postulated that the over-activation of Calpain-1 in these conditions may be a response aimed to control the damage rather than the cause of the cellular damage (21).

Certainly, *CAPN1* is involved in many essential neural processes and functional pathways common to corticospinal and cerebellar tracts and represents a link between hereditary ataxia and HSP, two groups of diseases considered separate so far (22). It is one of the so called “ataxia–HSP spectrum disease genes,” a group of more than 60 genes encoding proteins which share many physical interactions and form several highly connected “protein communities” (22). The three major functional clusters are lipid metabolic processes, acid metabolic processes and intracellular transport processes (22).

Particularly, *CAPN1* shares a role in regulation of the autophagy process with other 8 genes (*GFAP, NPC1, PSEN1, ARSA, PSAP, UCHL1, POLR3A, ATP13A2*), in cellular catabolic processes with 15 genes (*ABCD1, HEXA, UCHL1, MTPAP, EXOSC3, GAN, ATP13A2, STUB1, PNPLA6, GLB1, AUH, GBA2, PSEN1, GALC, ABHD12*) and in protein maturation processes with 4 genes (*FXN, PSEN1, AFG3L2, STUB1*) (22).

Given these premises, it seems obvious that mutations in this gene may lead to a complex spastic- ataxia phenotype with early onset (16, 23–26).

However, this assumption was recently challenged by the report of few patients with an involvement limited to the corticospinal tract: in one case, a congenital-onset pure HSP and, in the other two subjects, an adult-onset spastic paraparesis with no additional symptoms (17, 18).

Our study supports these recent observations by identifying a HSP phenotype with no cerebellar signs in a novel *CAPN1*-mutated family. The c.959delA mutation here reported is a novel variant which causes protein truncation within the CysPC domain with loss of the Ca binding domains located downstream, thereby leading to a likely loss of protein function.

As for many other widely expressed genes, the reason why the molecular defects in *CAPN1* lead to a clinical expression confined to a single system in some patients remains unknown.

A role for the location of the mutations seems unlikely. In fact, hot spot regions were not observed so far and the mutations spread along the entire gene irrespectively of the phenotype (Table 1) although a higher concentration is observed in the first two thirds of the protein corresponding to the protease domains.

**Table 1:** List of the variants described so far. Mutations spread along the entire gene irrespectively of the phenotype.

**PURE HSP**	**ATAXIA/HSP**	**HSP/OTHER SYSTEM INVOLVEMENT**
c.221G>A	c.1534C>T	c.2118+1G>T
c.397C>T	c.759+1G	c.884G>C
c.911C>T	c994G>A	c.1579C>T
c.1418G>T	c1176G>A	c.1579C>T
	c.843+1G>C	
	c.183dupC	
	1534C>T	
	c.C463T	
	c.C1142T	
	c.1579C>T	
	c.406delC	
	c.1605+5G>A	

Although our patients are adult subjects, we cannot fully exclude that they will develop other neurological system involvement later in the disease progression. However, it should be noted that the HSP-ataxia patients described so far presented with a complicated phenotype already at young age, thus suggesting an early complete expression of the molecular defect.

These findings allow us to confirm that the spectrum of phenotypes linked to mutations in *CAPN1* includes pure HSP with onset in adulthood.

This evidence, although limited to few cases so far, leads to relevant implications in genetic counseling and in genotype-phenotype correlation in *CAPN-1*-related disorders.

Further studies are mandatory in order to clarify the pathophysiological mechanisms underlying the different clinical expression of mutations in the same gene.

## Data Availability

This manuscript contains previously unpublished data. The name of the repository and accession number are not available.

## Ethics Statement

The study was conducted in a diagnostic setting and therefore does not require approval by the ethics committee.

## Author Contributions

SC: acquisition of data, analysis and interpretation of data, and drafting of manuscript. MB: acquisition of data, analysis and interpretation of data, and critical revision. AC, ST, SG, FC, EB, and AG: acquisition of data, analysis and interpretation of data. FM, FS, LS, and AP: analysis and interpretation of data and critical revision. MF: study conception and design, analysis and interpretation of data, and drafting of manuscript.

### Conflict of Interest Statement

The authors declare that the research was conducted in the absence of any commercial or financial relationships that could be construed as a potential conflict of interest.
