# Supplementary material for: A Novel CAPN1 Mutation Causes a Pure Hereditary Spastic Paraplegia in an Italian Family
Source: Front Neurol. 2019 Jun 5;10:580. doi: 10.3389/fneur.2019.00580 (PMC6560055; doi:10.3389/fneur.2019.00580)
Supplement: Supplementary file 1 [file Table_1.DOCX]

Gene list of the targeted gene panel

AARS, ABCD1, ADD3, AFG3L2, ALDH18A1, ALS2, AMPD2, ANO10, AP4B1, AP4E1, AP4M1, AP4S1, AP5Z1, APTX, ARHGEF10, ARL6IP1, ARSI, ASAH1, ATL1, ATL3, ATM, ATN1, ATP13A2, ATP1A2, ATXN10, B4GALNT1, BICD2, BSCL2, C10ORF2, C12ORF65, C19ORF12, CABC1, CACNA1A, CACNB4, CAPN1, CASK, CAV1, CCDC88C, CHCHD10, CHMP1A, COASY, CP, CPT1C, CYP27A1, CYP2U1, CYP7B1, DARS, DARS2, DCAF17, DCTN1, DDHD1, DDHD2, DGAT2, DNM2, DNMT1, DSTYK, DYNC1H1, EEF2, EGR2, ELOVL4, ELOVL5, ENTPD1, EPT1, ERLIN1, ERLIN2, EXOSC3, FA2H, FARS2, FGD4, FGF14, FIG4, FLRT1, FMR1, FTL, FXN, GAN, GARS, GBA2, GBE1, GDAP1, GJB1, GJC2, GRID2, GSN, HEXA, HEXB, HSPB1, HSPB3, HSPB8, HSPD1, IBA57, IFRD1, IGHMBP2, ITPR1, KANK1, KCNA1, KCNC3, KCND3, KIAA0196, KIAA0226, KIF1A, KIF1B, KIF1C, KIF5A, L1CAM, LITAF, LMNA, MAG, MARS, MARS2, MFN2, MME, MORC2, MPZ, MRE11A, MTHFR, MTPAP, MTTP, NARS2, NEFL, NEK1, NIPA1, NOP56, NPC1, NT5C2, OPA1, OPTN, PANK2, PDYN, PEX10, PEX7, PGAP1, PHF21A, PHYH, PLA2G6, PLP1, PMM2, PMP22, PNPLA6, POLG, PRKCG, PRRT2, PYGM, RAB3GAP2, RAB7A, RARS2, REEP1, REEP2, RNF170, RTN2, SACS, SARS2, SCP2, SEPSECS, SETX, SH3TC2, SIGMAR1, SIL1, SLC1A3, SLC33A1, SLC9A6, SOD1, SPAST, SPG11, SPG20, SPG21, SPG7, SPTBN2, STUB1, SYNE1, SYNE2, TBCD, TBCE, TBK1, TBP, TDP1, TECPR2, TFG, TGM6, TK2, TMEM240, TRIM2, TRMT5, TRPC3, TRPV4, TSEN2, TSEN34, TSEN54, TTBK2, TTPA, UBR4, USP8, VAMP1, VAPB, VCP, VPS37A, VRK1, WDR48, YARS, ZFR, ZFYVE26, ZFYVE27
